# A Review of Associations between Externalizing Behaviors and Prenatal Cannabis Exposure: Limitations & Future Directions

**DOI:** 10.3390/toxics10010017

**Published:** 2022-01-05

**Authors:** Ami S. Ikeda, Valerie S. Knopik, L. Cinnamon Bidwell, Stephanie H. Parade, Sherryl H. Goodman, Eugene K. Emory, Rohan H. C. Palmer

**Affiliations:** 1 Behavioral Genetics of Addiction Laboratory, Emory University, Atlanta, GA 30322, USA; 2 Department of Psychology, Emory University, Atlanta, GA 30322, USA; psysg@emory.edu (S.H.G.); eemory@emory.edu (E.K.E.); 3 Department of Human Development and Family Studies, College of Health and Human Sciences, Purdue University, West Lafayette, IN 47907, USA; vknopik@purdue.edu; 4 Institute of Cognitive Science, University of Colorado Boulder, Boulder, CO 80309, USA; lcb@colorado.edu; 5 Department of Psychology and Neuroscience, University of Colorado Boulder, Boulder, CO 80309, USA; 6 Department of Psychiatry and Human Behavior, Alpert Medical School of Brown University, Providence, RI 02903, USA; Stephanie_Parade@Brown.edu; 7 Bradley/Hasbro Children’s Research Center, E.P. Bradley Hospital, East Providence, RI 02915, USA

**Keywords:** prenatal cannabis exposure, substance use, adolescence, externalizing traits

## Abstract

In utero cannabis exposure can disrupt fetal development and increase risk for various behavioral disruptions, including hyperactivity, inattention, delinquent behaviors, and later substance abuse, among others. This review summarizes the findings from contemporary investigations linking prenatal cannabis exposure to the development of psychopathology and identifies the limitations within the literature, which constrain our interpretations and generalizability. These limitations include a lack of genetic/familial control for confounding and limited data examining real world products, the full range of cannabinoids, and motives for use specifically in pregnant women. Taken together, our review reveals the need to continue to improve upon study designs in order to allow researchers to accurately draw conclusions about the development of behavioral consequences of prenatal cannabis exposure. Findings from such studies would inform policy and practices regarding cannabis use during pregnancy and move the field toward developing a comprehensive teratogenic profile of cannabis similar to what is characterized in the prenatal alcohol and tobacco literature.

## 1. Introduction

Cannabis use is increasing, including among pregnant women. However, there is still a tremendous gap in the teratogenic profile of cannabis and its impact on various aspects of health. Many women believe there is minimal risk associated with cannabis use [1,2]. Overall, the observed rates of cannabis use among women of childbearing age and during pregnancy have varied from 4.0–18.0%. The 2007–2012 National Survey on Drug Use and Health (NSDUH) data revealed that among pregnant women who used cannabis within the past year, 16.2% reported smoking almost daily compared to 12.8% reported by nonpregnant women. Of these same women, 18.1% met criteria for cannabis dependence, while only 11.4% of nonpregnant women met these criteria [1]. Among women of childbearing age, 11.5% reported using cannabis within the past month and 17.5% within the past year [3]. Notably, other studies have reported similar findings [4,5]. Since 2012, rates of use during pregnancy have varied with the 2016–2017 NSDUH report finding 7.0% of women using cannabis while pregnant, with even higher rates of use (12.1%) occurring during the first trimester [5]. In the 2018 NSDUH report, slightly fewer women (4.7%) reported using cannabis while pregnant within the past month [3]. However, more recent data from the 2019 NSDUH report found that 5.4% of women reported using cannabis while pregnant in the past month, with the highest rate of use occurring within the first trimester (9.1%) and the lowest rate of use occurring within the third trimester (3.3%) [3]. As demonstrated by these prevalence estimates, cannabis use during pregnancy continues to be a public health concern.

Delta-9-tetrahydrocannabinol (THC), the main psychoactive component in cannabis, remains a compound of high concern for pregnant women because it is a highly lipid soluble substance that is readily absorbed through the lungs, liver, and adipose tissues [6,7]. The highly lipid soluble nature of THC allows it to pass through the blood–brain barrier and placental membrane with ease [8]. In the human body, response to THC is mediated by the endocannabinoid system. The endocannabinoid receptor system is an important regulatory mechanism that involves endocannabinoids, which are endogenous lipids also known as anandamides, enzymes, and two well studied cannabinoid receptors—cannabinoid receptor type 1 (CB1) and cannabinoid receptor type 2 (CB2). The CB1 and CB2 receptors are two G protein coupled receptors [9], which are widely distributed throughout the central nervous system and are even found in some peripheral tissues [9]. CB2 receptors are mainly found in immune cells [10] and are also present on dopamine neurons within the ventral tegmental area (VTA), which plays a crucial role in the brain’s reward pathway [11]. Additionally, immunohistochemistry analyses have shown the presence of CB1 receptors in all layers of the human placental membrane [12]. This is of particular interest within the current review as cannabis use during pregnancy has been found to result in heavier placental weight, which could be a result of chronic hypoxia [13], although the actual impact that this may have on the fetus and the pregnancy are unknown.

Other roles that the endocannabinoid system is suspected to be involved in include synaptic formation, neurogenesis [14], implantation, embryo development, immune regulation [15] and synaptic plasticity [16]. Importantly, by 14 weeks of gestation, the endocannabinoid receptor system has matured in the brain, and, by 17–18 weeks of gestation, mature adult-like receptor levels can be seen in the globus pallidus [17]. Based on this knowledge, several studies have investigated the association between early cannabis exposure (in utero) and neuronal functioning. In a study of human fetal brain tissues collected at 18–22 weeks of gestation, DiNieri et al. [18] revealed a negative correlation between in utero cannabis exposure and dopamine messenger RNA (mRNA) expression in the nucleus accumbens [18]. Cannabinoids have also been found to increase dopamine function and synthesis, as well as inhibit dopamine reuptake in important reward related brain regions [19]. In an early study, a sex-dependent reduction in dopamine mRNA expression levels was also observed in human fetal brain tissue in the basolateral amygdala, predominately in males [20], suggesting a vulnerability in dopamine activity in important areas of the brain connected to reward processing and potential sex effects that require further investigation. Both the presence of CB1 receptors in the central nervous system and placental membrane, as well as the maturation of the endocannabinoid receptor system as early as 14 weeks of gestation, underscores the endocannabinoid systems significance in human development.

The current scoping review examines the published literature on the effects of maternal cannabis during pregnancy (MCDP). Specifically, we describe the neurodevelopmental consequences of MCDP with a focus on subsequent substance use and the associated externalizing behaviors during childhood and notably adolescence, which is both a significant period of brain refinement that is characterized by synaptic pruning and increased myelination [21], and early substance use initiation during this time period poses as a risk factor for later substance use [22,23,24,25]. Additionally, significant molecular and synaptic changes have been found in dopamine neurons in the VTA [26] as a consequence of MCDP, and this may serve as yet another potential risk factor for future substance use, though no causal conclusion can be determined.

## 2. Motivations for This Review: A Changing Landscape of Cannabis Potency

In contrast to the literature on fetal exposure to mothers’ tobacco smoking, research on the effects of MCDP has been limited. However, recent shifts in the legalization, routes of administration, motives for using cannabis, and pharmacological profile of cannabis sativa support the need for the continued study of MCDP to fill emerging gaps in knowledge.

First, the legalization of cannabis has rapidly increased across the United States, with cannabis being recreationally available in 17 states and medicinally available in 38. This widespread legalization may be associated with increased cannabis use by pregnant women. Indeed, cross-sectional data from the 2016 Pregnancy Risk Assessment Monitoring System (PRAMS) revealed that women from states with legalized recreational cannabis were more likely to use before, during, and after pregnancy compared to women in states where recreational cannabis use is illegal [27]. However, it should be noted that women living in states where recreational cannabis use is illegal may be more hesitant to report accurate cannabis use. As such, additional research is still needed to substantiate these trends. Importantly, many states that legalized cannabis prior to others had higher prevalence of cannabis use prior to legalization, which makes it difficult to infer a direct effect of legalization. Still, the shift in use trends by pregnant women cannot be ignored.

Second, along with important changes in legalization, we must also consider the various modes of administration women are using during pregnancy. A recent pilot study, spearheaded by Kaiser Permanente, Northern California medical centers, collected self-report measures from women the year prior to pregnancy and during pregnancy (~8 weeks of gestation). Among the women that endorsed using cannabis during their pregnancy, 42.1% reported smoking, 15.8% vaped, 15.8% used edibles, and 5.3% used lotions; of the women that reported the route of administration, 84.6% only used one mode of administration and 15.4% reported two modes [28]. Unfortunately, the current study failed to report cannabis potency or cannabis strains, which differs across users and routes of administration that can differentially impact fetal development. Finally, smoking was most commonly associated with daily use. Interestingly, the route of administration before pregnancy did not accurately represent the route of administration used during pregnancy [28], suggesting that drug use history may inform chronicity of use, but not administration and cannabis potency during pregnancy.

Third, although legalization suggests some explanation for shifting use trends, shifts in motives also support the need for better understanding the consequences of MCDP. Yet, few have investigated motives to use in pregnant women. A 2017 PRAMS survey found that women reported using cannabis during their pregnancy as a way to reduce stress and/or anxiety, manage nausea or vomiting, relieve pain, have fun or relax, and to relieve chronic condition symptoms [29]. These findings support the idea of both medical and recreation motives for cannabis use during pregnancy. However, given that studies of motives for cannabis use among clinical populations [30,31,32] have largely excluded pregnant women; this remains an important, understudied area of investigation.

Fourth, the past few decades have been characterized by drastic changes in cannabis potency and changes in proportion of THC:CBD levels. As a result, findings from older studies may no longer apply. For example, average THC:CBD ratios have increased from 23:1 to 104:1, which reflects the growing popularity of consuming higher concentrations of THC [33]. It is suggested that THC with low CBD may lead to greater intoxication especially in infrequent users while THC with high CBD may lead to reduced intoxication [34]. This may suggest that CBD acts as an inverse agonist that reduces the effect of THC. In general, it has also been found that CBD has a lower affinity to the main cannabinoid receptors compared to THC [31]. Further, in the United States, between 2008 to 2017, average THC concentrations have increased from 8.9% to 17.1% [33]. Additionally, a recent meta-analysis reported that, between 1970–2017, THC concentrations in concentrates have increased, each year, by 0.57% [35]. An example of the alarming potency of concentrates was reported in a 2015 study, which found concentrate (hash, hashish oil, wax, or cartridges) potency was on average 62.1% THC [36]. Overall, these changing trends support the importance of improving the understanding of the types and potency of products used by pregnant women and whether the risk profile to the fetus differs across these different cannabinoids, THC:CBD proportions, and formulations.

Despite the evidence for these three trends, in our survey of the literature, few studies report on legal status, motives for use, or potency, leaving significant gaps in the literature. As just one example, there are increasingly different forms of cannabis and different potencies available that may increase risk to pregnant users, but which have not been systematically evaluated. Rather, studies have mainly focused on the impact THC has on the developing fetus; however, widely available products include THC, CBD, or both at varying levels. Importantly, CBD products available on the market today may be vulnerable to mislabeling. For example, in an examination of 84 cannabidiol samples, 18 contained up to 6.43 mg/mL of THC [37]. This further supports the need to systematically evaluate the products mothers are currently using. Traditionally, animal models have also played an integral role in elucidating the direct consequences of dosage and timing in prenatal studies; unfortunately, the classification of cannabis as a Schedule 1 drug has limited researchers to use THC at lower potency levels and variable THC:CBD ratios [38] than what is currently available on the market. Therefore, the translational power is low. However, this does not discount the information that can be gathered from preclinical studies including transmission to the fetus and clarifying the possible differential effects of CBD and THC.

Given these trends in legalization, motivation to use, cannabis potency, and THC:CBD ratios, it is essential to better understand cannabis use among pregnant women and their offspring’s subsequent substance use and associated externalizing behavior. As a step in that direction, we reviewed the recent literature on the teratology of cannabis and its impact on childhood and adolescent substance use and associated externalizing behaviors. We identified articles using PubMed, PsycINFO, and Google Scholar. Inclusion criteria included empirical studies, review articles, and meta-analyses examining prenatal cannabis exposure with externalizing behaviors and substance use. Studies were excluded if prenatal cannabis exposure was controlled for with no examination of its direct effect or studies examining behaviors other than externalizing traits. Our search strategy involved using a combination of search terms including, but not limited to, “prenatal cannabis”, “substance use”, “externalizing traits”, “childhood outcomes” and “developmental psychopathology”. Specifically, the following terms encompassed “prenatal cannabis/marijuana, in utero cannabis/marijuana, maternal cannabis/marijuana”, “substance use, alcohol use, cannabis use, tobacco use, drug use, substance misuse, substance experimentation”, “externalizing traits, aggressive behavior, inattention, hyperactivity, delinquency, executive dysfunction” and “developmental psychopathology, neurodevelopment, cognitive development, behavioral development” while using “AND” and “OR” Boolean operators. Once all articles were identified, two undergraduate students and a graduate student reviewed the literature, summarized the findings, and built a table including all articles. Further, we obtained references from review articles and related preclinical studies.

## 3. MCDP Effects on Offspring Development of Externalizing Behaviors

We identified 19 studies that have examined the effect of MCDP and a multitude of behavioral and cognitive outcomes, including executive dysfunction, inattention, and hyperactivity (for a summary of the findings please see Table A1 in Appendix A). Much of our understanding is based on data from the following three large, longitudinal studies: the Maternal Health Practices and Child Development Project (MHPCD) [39], the Ottawa Prenatal Prospective Study (OPPS) [40] and Generation R [41]. One of the advantages of these prospective, longitudinal designs beginning in pregnancy and following offspring through childhood is that they gathered data on prenatal cannabis use at multiple intervals during pregnancy, reflecting use specific to the 1st, 2nd, or 3rd trimester, or throughout pregnancy. Studies centered on the 1st trimester define it as the first 13 weeks of pregnancy during which important events such as gastrulation, neural tube formation and head formation occur [42]. Generally, trimesters (i.e., 1st, 2nd, 3rd trimesters) reflect both the timing and/or duration of exposure, with increased exposure occurring across pregnancy (i.e., 1st vs. 2nd and 3rd trimesters).

Several of the studies examined fetal cannabis exposure in relation to externalizing behavior problems in offspring, which is defined as aggressive, rule-breaking behavior, and delinquent behavior [43]. Across studies, results support associations between fetal exposure regardless of duration and offspring’s later inattention and delinquency behavior problems [44]. Heavy exposure, defined as more than ~1 “joint” per day, during the first trimester resulted in significantly higher scores on the delinquency scale (delinquent behavior, aggressive behavior, and externalizing problems) as measured by the mothers’ report on the Child Behavior Checklist (CBCL) [44]. Additionally, adolescents were twice as likely to display delinquent behaviors compared to adolescents with no exposure or exposed to less cannabis prenatally [45].

Similarly, inattention may be associated with any MCDP regardless of duration of exposure, as 1st trimester exposure was a significant predictor of inattention at the age of 10, as measured by the Swanson, Noland, and Pelham (SNAP) checklist [44]. Poorer performance on a task measuring sustained attention was observed in 6-year-old children exposed to cannabis prenatally [46], and poorer stability performance (how stable one’s attention is during a task) was found in adolescents 13–16 years of age with heavy prenatal cannabis exposure (defined as greater than or equal to 6 joints a week) [47]. Consequently, rodent models suggest a relationship between MCDP and altered neuronal excitability and synaptic plasticity in the prefrontal cortex [48]. This would be especially relevant to clinical populations as several neuropsychiatric disorders have been linked to disruptions in prefrontal cortex function. For example, neurobehavioral deficits such as inattention and delinquent behavior have been linked to prefrontal cortex differences [49]. One study reported that 18–22-year-old participants with prenatal cannabis exposure displayed more commission errors, a measure of impulsivity, and this deficit was reflected in increased activity in the bilateral prefrontal cortex, which could be explained as an increased effort to perform the task at hand [50].

In contrast, impulsivity may be a behavioral outcome unique to prolonged duration of exposure. Second trimester exposure has been associated with increased impulsivity [51], which was also reported by Leech et al. [52]. Six-year-old children with 2nd trimester exposure displayed increased deficits related to impulsivity compared to inattentiveness [52]. While combined 2nd and 3rd trimester exposure has been associated with increased hyperactivity, inattention, and impulsivity [44].

Furthermore, MCDP can lead to secondary problems including school difficulties. Deficits in school performance were found to be mediated by attention problems, depressive symptoms, and early initiation of cannabis [53]. MCDP has also been associated with increased rates of autism spectrum disorders, learning disorders, ADHD and conduct disorders [54]. Previous studies have also linked MCDP to increased aggressive and inattentive behaviors in girls but not boys, as early as 18 months of age [55]. Additionally, exposed children display increased behavioral problems [56] and increased externalizing and attention problems [57]. Nevertheless, findings are mixed as others have reported no relationship between MCDP and externalizing behaviors [58]. Relatedly, a new study is emerging with promising objectives to examine the role MCDP plays on aggression and executive function in children 3 ½ to 7 years of age [59]. This more recent study is unique as it focuses on early life outcomes when interventions may be more successful. However, this study, like others, was unable to collect information on cannabis potency, quantity, time of exposure, strain or product type and will be completed retrospectively.

As evidenced by the reported studies much of the data available examining consequences of MCDP occurred prior to legalization and reflect potency levels significantly lower than products currently available. Newer studies are required to draw meaningful conclusions about MCDP consequences at current potency levels, strains, and routes of administration. Additionally, study measurements should capture duration of use as neurobehavioral differences may emerge as a result of prolonged use versus throughout pregnancy.

## 4. MCDP Effects on Offspring Development of Substance Use

Given that MCDP may exacerbate childhood behavioral problems and the fact that externalizing problems are associated with early substance use initiation [60], we examined the literature for studies of prenatal cannabis exposure and offspring subsequent substance use. Childhood externalizing behaviors have been theorized as an intermediary phenotype linking prenatal cannabis and alcohol exposure to daily drug use (i.e., indirect effect) later in life [61]. It also appears that the relationship among these variables may be cyclical as externalizing traits are positively associated with substance use, but results have also shown that using substances of abuse may lead to increased externalizing behaviors [62]. This cyclical model is similar to those represented in the FASD [63] and alcohol use disorder literature [64].

Identification of risk factors for early substance use initiation is critical as early initiation of cannabis use may be a risk factor for the development of cannabis use disorder (CUD) later in life. For example, cannabis use is associated with a 1.6-fold increase in the odds of being diagnosed with a CUD by 22 years of age in comparison to those that do not begin using cannabis early in life [65]. Additionally, adolescents, 14 years of age, exposed to cannabis prenatally are more likely to initiate cannabis use sooner, use cannabis more frequently than adolescents with no exposure during pregnancy [66], and continue to use cannabis later in life [67]. Other studies revealed that these associations may be specific to males and not females [68], but additional replication studies are needed.

Although important associations have been drawn from the longitudinal studies described earlier, preclinical studies also provide important insight into the impact prenatal cannabis exposure has on subsequent substance use. Specifically, preclinical studies provide an important first step towards understanding the complex relationship between MCDP and subsequent substance use. For example, rats prenatally exposed to cannabis did not self-administer heroin more than control rats; however, rats with prenatal cannabis exposure did display higher heroin-seeking behaviors when exposed to mild stress, which suggests an altered motivation for drug use [69]. Again, findings are mixed as rats prenatally exposed to cannabis and control rats did not differ in their ethanol self-administration, and ethanol-seeking behavior was not affected by mild stress [70]. However, female rats prenatally exposed to cannabis displayed increased acquisition of morphine self-administration, which corresponded with increased µ opioid receptor density in the prefrontal cortex, hippocampus, amygdala, ventral tegmental area and the periaqueductal grey matter [71]. As previously mentioned, this is significant as the ventral tegmental area plays an important role in the reward pathway. For a comprehensive review of the preclinical findings see Campolongo et al. [72]. Finally, genetic variations are important in examining the association between prenatal cannabis exposure and the traits presented within the review, and while these behavioral changes could be associated with shared genetic effects, this has yet to be shown. For a review on multigenerational transmission see Szutorisz and Hurd [73], and for a review on epigenetic pathways associated with psychiatric diseases see Smith et al. [74]. Overall, understanding the consequences of MCDP seems to be an important first step that would aid in the development of potential interventions that could help circumvent later substance abuse.

## 5. Future Directions and Conclusions

As rates of cannabis use during pregnancy increase and given the heterogeneity that exists in cannabis use behavior, studies should consistently collect information on cannabis potency, strains, routes of administration, motivation to use both THC and CBD, and chronicity of use. The collection of these critical variables will begin the necessary process of elucidating the relationship between MCDP and the resulting neurobehavioral consequences. Furthermore, while in utero cannabis exposure appears to negatively impact behavioral development, it may be that these deficits are exacerbated by the duration of exposure (1st, 2nd, 3rd trimester or throughout pregnancy). Continued investigation and refinement of current methodologies is needed to elucidate the role of MCDP on fetal development and neurobehavioral outcomes.

Additionally, with continued changes in products and potency of cannabis available, the gaps that exist within our current knowledge continue to increase. Importantly, the proposed challenges are not unique to prenatal cannabis studies; they also characterize much of the prenatal alcohol [75] and tobacco [76] literature. Nonetheless, it is essential to quantify reliable and specific effects of prenatal cannabis exposure that account for potential confounders. Specific to cannabis use, it is essential to account for various cannabinoids and potencies. Should quantifiable and specific effects be identified for prenatal exposure to specific forms and potencies of cannabis, this would allow for more specific guidelines related to cannabis use during pregnancy and may lead to the development of a teratogenic profile. This teratogenic profile can be used to develop standard diagnostic criteria, similar to facial dysmorphologies and neurobehavioral deficits captured for fetal alcohol spectrum disorders [77,78,79,80,81,82,83], which could inform formal guidelines and preventive care for the fetus. Overall, improvements in study design are needed to be able to infer causality and assess the true teratogenic impact of cannabis. Thus, we have identified four gaps within the field and provide the necessary recommendations.

First, significant changes in cannabis potency have occurred over the past decade, resulting in disparate neurobehavioral outcomes and data that does not reflect the current potency, strains or products available in the market today. This can be improved by utilizing standardized reporting methods. New studies should examine how the full range of cannabinoids, potency, frequency of use, strain, method of administration, and motivation for use impacts fetal development and behavior as the effects may vary across individuals. Specifically, given the nuance associated with accurately capturing a mother’s experience during pregnancy, secondary objective measures should be incorporated in future study designs. In addition to self-report measures, future studies should collect cannabis product samples directly from the mother for further quantification of cannabinoids (e.g., THC vs. CBD vs. THC:CBD ratios), strains, and potency. Future study designs should also incorporate objective biological assays to supplement the self-reporting of cannabis use during pregnancy; these can include but are not limited to urine screens, neonatal meconium, or umbilical cord tissue. Previous studies support a possible underreporting of prenatal cannabis use and emphasize the need for both self-report and biological assay measures [84,85]. While an objective secondary measure for frequency of use, methods of administration, and motivation may not currently exist we can utilize preclinical studies to bolster our understanding of the impact frequency and route of administration have on transmission to the fetus and impacts on development. Preclinical studies may even indirectly address the impact of motivation (via product choice) on fetal development by examining in utero transmission of THC vs. CBD to the fetus and the associated consequences. These necessary measures could provide important insight into the consequences of prenatal cannabis exposure at varying toxicity levels and at varying windows of development. More importantly, we can begin to disentangle the differences between cannabis use disorder users vs. medically motivated first time users, THC vs. CBD users, and the behavioral health outcomes in chronic and acute users [86] with the collection of more robust data sets.

Second, identifying motives for use and other maternal risk data could inform intervention, preventive care, and targeted treatment for mothers who may be at increased risk for using during their pregnancy. Currently, younger age women between the ages of 16–24 report higher cannabis use both before and during pregnancy compared to women between the ages of 25–45. In addition to younger maternal age, other potential risk factors include unplanned pregnancies and prior drug use including alcohol and tobacco use [28], although others remain unidentified. Additionally, upon identifying potential maternal risk factors, studies should control for these as well as other substances or other risky behaviors in which pregnant women may engage. Few studies currently exist that examine motives and other potential risk factors for use in pregnant women despite the implications this has for both the mother and fetus.

Third, the NSDUH has reported increased rates of co-occurring substance use among a sample of pregnant and nonpregnant women. The data reflects a 2–3 times increased risk of using cannabis in women who used other drugs of abuse such as tobacco, alcohol, and other illicit drugs [1,87]. Thus, it is necessary to understand the impact co-occurring substance use has on behavioral development as both a maternal risk factor and additive teratogen.

Finally, and most importantly, given the overall paucity of well-controlled studies that account for shared genes and environment, no causal conclusions can be drawn at this time. For example, findings have shown that prenatal exposure as well as maternal cannabis use prior to pregnancy was associated with increased externalizing problems [43]. However, this effect was also observed with paternal cannabis use, which suggests a potential gene environmental correlation [43]. Similar to previous suggestions, study designs should incorporate multimethod measures, although there has been agreement between parent and teacher behavioral ratings in children with prenatal alcohol exposure [88]. Furthermore, future study designs may want to examine fetal behavioral profiles in response to external stimulation with the intention of obtaining an objective measure of prenatal behavior, linking behavioral response rates to in utero cannabis exposure, and obtaining a behavioral foundation from fetal responses that have continuity with aspects of postnatal externalizing traits (e.g., faster response time, higher rates of behavior, or prolonged responding). For examples of such methods please see DiPietro [89] and Emory [90]. Additionally, despite findings suggesting that there may be an association between MCDP and subsequent substance abuse, the potential genetic contribution cannot be ignored. We need to cultivate data that controls for shared genes and environment and other confounders, such as parenting behavior, familial psychopathology, family functioning, overall quality of home environment and environmental enrichments, to establish a causal relationship and critically examine the impact cannabis has on neurobehavioral consequences. This can be achieved through discordant sibling studies, which would enable researchers to quantify the specific effects of the shared genes and environment.

Overall, new robust data sets are needed in order to draw meaningful causal conclusions that reflect the current market of cannabis products being used by pregnant women. This requires the refinement of measures and methodological practices as well as the inclusion of proper controls. Well-designed studies with comprehensive measures that quantify specific and reliable effects will begin informing the development of a teratogenic profile and address the gaps in our understanding of the complex relationship between prenatal cannabis exposure, substance use, and development as a whole.

## Data Availability

Not applicable.

## Appendix A

**Table A1:** Reported Effects of Prenatal Cannabis Use on Developmental Outcomes.

Study	Sample	Sample Makeup	Duration of Exposure		Secondary Problems + Substance Use	Reported Relationship
			Regardless of Duration	Prolonged Exposure		
[56]	OPPS	*N* = 56			Increased behavioral problems (6–9 years).	ηp^2^ = 0.12, *p* = 0.01
[46]	OPPS	*N* = 126	Poorer sustained attention. More omission errors (6 years).			ηp^2^ = 0.07, *p* < 0.05
[47]	OPPS	*N* = 152	Poorer attentional stability and less consistent reaction time (13 to 16 years).			ηp^2^ = 0.07 *p* < 0.01
[50]	OPPS	*N* = 35		Increased comission errors.		ηp^2^ = 0.17, *p* < 0.02
[91]	OPPS	*N* = 145			Poorer response times (13 to 16 years).	
[52]	Maternal Health Practices and Child Development (MHPCD) Study	*N* = 608		Increased impulsivity. Fewer errors of omission (6 years).		B = −0.56, *p* < 0.05
[44]	MHPCD	*N* = 635 mother reports and 575 teacher reports	Increased inattention. Increased delinquency and externalizing problems (10 years).	Increased impulsivity and hyperactivity.		ηp^2^ = 0.02, *p* = 0.005 ηp^2^ = 0.01, *p* = 0.01 ηp^2^ = 0.01, *p* = 0.01
[66]	MHPCD	*N* = 563			Early onset and frequency of marijuana use (14 years).	B = 0.13, *p* = 0.04 B = 0.25, *p* = 0.02
[45]	MHPCD	*N* = 525	Increased delinquent behavior (14 years).			OR = 1.84, *p <* 0.03
[51]	MHPCD	*N* = 636		Increased impuslivity		B = 1.86, *p* = 0.01
[53]	MHPCD	*N* = 524			Lower performance on intelligence tests that was mediated by inattention, depressive symptoms and early substance use (14 years).	*b* = 0.45, *p* < 0.0005 *b* = −0.11, *p* < 0.005 *b* = 0.08, *p* < 0.05
[92]	MHPCD	*N* = 608			Early onset marijuana use (15 years and again at 22 years).	OR = 1.40, *p* = 0.001
[55]	Generation R	*N* = 4077			Increased aggressive behavior. Increased inattention (18-month-old girls only).	*b* = 2.02, *p* = 0.02 *b* = 1.04, *p* < 0.001
[43]	Generation R	*N* = 5903			Increased externalizing problems (7–10 years).	B = 0.53, *p* < 0.001
[58]	Miami Prenatal Cocaine Study	*N* = 407			No differences in externalizing or internalizing behaviors.	
[57]	Adolescent Brain and Cognitive Development (ABCD) Study	*N* = 11489			Increased externalizing and attention problems (9 to 11 years).	*b* = 1.09, *p* = 0.05 *b* = 2.05, *p* < 0.001

ηp^2^ = Partial eta-squared (calculated using the reported *F*-value and degrees of freedom provided in the respective papers). OR = Odds ratio. B = Unstandardized beta coefficient. *b* = Standardized beta coefficient.
